# Comparison of Long-Term Clinical Outcomes Between Immediate-Release and Slow-Release Carvedilol: A National Real-World Database Analysis

**DOI:** 10.3390/jcm15041417

**Published:** 2026-02-11

**Authors:** Hack-Lyoung Kim, Hyun Sung Joh, Sang-Hyun Kim

**Affiliations:** Division of Cardiology, Department of Internal Medicine, Seoul Metropolitan Government-Seoul National University Boramae Medical Center, Seoul National University College of Medicine, 5 Boramae-ro, Dongjak-gu, Seoul 07061, Republic of Korea; wingx4@naver.com (H.S.J.);

**Keywords:** carvedilol, major adverse cardiac events, medication adherence, prognosis

## Abstract

**Background:** Carvedilol, a non-selective β- and α1-blocker, is available in immediate-release (IR) and slow-release (SR) formulations. While carvedilol IR requires twice-daily dosing, SR was developed to improve adherence by allowing once-daily administration. This study aimed to compare long-term clinical outcomes between SR and IR carvedilol. **Methods:** A total of 38,563 patients taking either SR (*n* = 6223) or IR carvedilol (*n* = 32,340) were retrospectively analyzed using national claims data. Baseline characteristics, medication adherence, and cardiovascular outcomes were evaluated over a median follow-up period of 730 days. Multivariable Cox regression analyses were performed to adjust for potential confounders. **Results:** Despite a higher burden of comorbidities in the SR group, patients taking SR carvedilol had significantly lower risks of major adverse cardiovascular events (MACE), non-fatal myocardial infarction, and heart failure hospitalization compared to those on IR carvedilol. The adjusted hazard ratio for MACE with SR compared to IR was 0.61 (95% CI, 0.556–0.670; *p* < 0.001). No significant difference in non-fatal stroke was observed between the groups. **Conclusions:** In this claims-based observational cohort, SR carvedilol was associated with more favorable long-term cardiovascular outcomes than IR carvedilol. However, this association does not establish causal superiority, and prospective randomized studies are needed to confirm these findings.

## 1. Introduction

Carvedilol, a non-selective β-blocker with additional α1-blocking activity, is extensively prescribed for the management of hypertension, congestive heart failure, and ischemic heart disease [1,2,3,4,5,6,7,8]. Its effectiveness in reducing morbidity and mortality across various cardiovascular conditions has been well-documented in the medical literature [2,5]. The pharmacokinetics of carvedilol significantly influence its clinical efficacy and patient compliance, both crucial in chronic conditions requiring long-term management [7].

Immediate-release (IR) formulations of carvedilol provide rapid absorption and a rapid onset of action, which are advantageous for acute management but may require multiple daily doses due to their short half-life [9,10]. This dosing frequency can lead to variability in plasma drug levels, potentially affecting the therapeutic consistency and increasing the risk of side effects. Additionally, taking medication multiple times a day can decrease patient compliance, which in turn may increase the risk of clinical events [10,11]. On the other hand, slow-release (SR) formulations are designed to maintain more stable plasma levels, enhance patient adherence, and potentially minimize drug-related adverse effects by extending the dosing interval [9,10].

Despite the pharmacological advantages of SR formulations, data comparing long-term outcomes between IR and SR carvedilol remain sparse. Previous studies have primarily focused on pharmacokinetic profiles, surrogate functional parameters (e.g., changes in left ventricular function) [12], medication adherence [10], and quality of life [13] associated with once-daily versus twice-daily dosing. A small number of prospective randomized trials in heart failure populations have evaluated non-inferiority between SR and IR formulations [14]; however, these studies were relatively limited in sample size, duration, or endpoint scope and therefore provide insufficient evidence to determine differences in major cardiovascular events. Consequently, large-scale real-world data comparing hard clinical outcomes between SR and IR carvedilol are still lacking. This knowledge gap hinders optimal therapeutic decision-making, particularly in chronic cardiovascular management, where sustained drug action and patient compliance are pivotal. This study aims to address this gap by comparing long-term clinical outcomes between IR and SR carvedilol. Through this comparison, we aim to provide evidence-based insights to guide clinical practice in selecting the appropriate formulation based on patient-specific conditions and overall treatment goals. We hypothesize that the SR formulation of carvedilol will yield superior long-term cardiovascular outcomes owing to improved pharmacokinetic properties and greater patient adherence.

## 2. Materials and Methods

### 2.1. Study Patients

In this study, we utilized data from the National Health Insurance Service-National Health Information Database (NHIS-NHID) of the Republic of Korea. Established in 2012, the NHIS-NHID is a comprehensive, government-maintained health database that collects data from all individuals enrolled in the nation’s mandatory health insurance program, which covers over 97% of the population. The database contains extensive health-related data derived from medical claims, including diagnoses coded according to the International Classification of Diseases (ICD), prescribed medications, medical procedures, and detailed patient demographics such as age, sex, income level, and region of residence. It also incorporates data on regular health examinations, providing valuable insights into preventive health behaviors. The breadth and longitudinal nature of the NHIS-NHID make it an invaluable resource for epidemiological and health outcomes research. In this study, the availability of such comprehensive data enabled an in-depth analysis of long-term clinical outcomes and healthcare utilization [12,13], thereby enhancing the validity of our findings by capturing a wide range of variables influencing health across the Republic of Korea.

**Figure 1:**
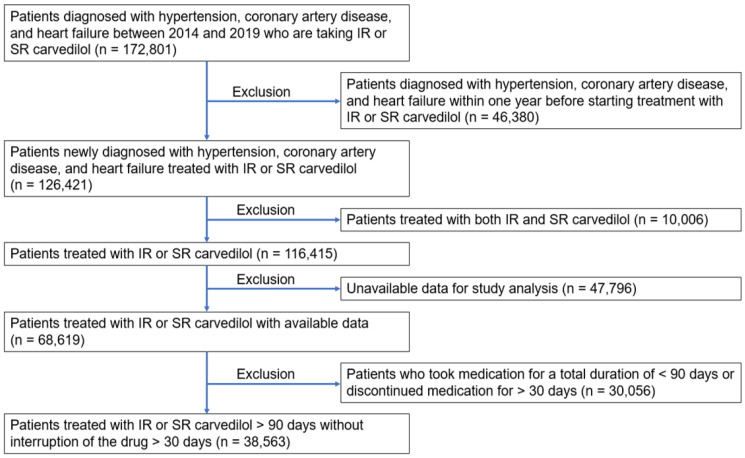
Flow chart of study enrollment. IR, immediate release; SR, slow release.

We initially screened 172,801 patients diagnosed with hypertension, coronary artery disease, and heart failure between 2014 and 2019 who were taking either IR or SR carvedilol. Subsequently, we excluded 46,380 patients who had already been diagnosed with hypertension, coronary artery disease, and heart failure one year prior to the initiation of carvedilol IR or SR. Therefore, from the remaining 126,421 patients newly diagnosed with these conditions during the specified period, we excluded 10,006 patients who had a history of taking both carvedilol IR and SR, as well as 47,796 patients lacking key variables for the study analysis, leaving a total of 68,619 patients. Finally, we excluded 30,056 patients with a total medication duration of less than 90 days or with a medication discontinuation period longer than 30 days. Thus, this study analyzed 38,563 patients. The flow chart for study enrollment is shown in Figure 1.

This research received approval from the Institutional Review Board (IRB) of the Korea NHIS (research management number NHIS-2023-1-585) and from the Seoul National University Boramae Medical Center (IRB number 07-2022-41). The study used a database provided by the NHIS, leading the IRB of Seoul National University Boramae Medical Center to waive the informed consent requirement. All procedures were carried out in accordance with the applicable guidelines and regulations.

### 2.2. Collection of Clinical Data

Body mass index was calculated as body weight (kg) divided by height squared (m^2^). Systolic and diastolic blood pressures were measured using an oscillometric device on the right upper arm. Individuals who smoked regularly within the past year were considered current smokers. Stroke, diabetes mellitus, dyslipidemia, coronary artery disease, myocardial infarction, and heart failure were diagnosed based on ICD-10 codes. Income level was determined from payments into the national insurance system and stratified into four quartiles. After an overnight 12-h fast, blood levels of the following parameters were measured: total cholesterol, low-density lipoprotein (LDL) cholesterol, high-density lipoprotein (HDL) cholesterol, triglycerides, glucose, and hemoglobin.

### 2.3. Clinical Events

The composite endpoint in this study was considered as major adverse cardiovascular events (MACE), which included death, non-fatal myocardial infarction, non-fatal stroke, and heart failure requiring hospitalization. Clinical events were identified using ICD-10 codes registered in the Korean National Health Insurance Service System (KNHISS) for reimbursement purposes at hospital discharge (I21-23 for myocardial infarction, I60-69 for stroke, including both hemorrhagic and ischemic strokes, and I50 for heart failure). Additionally, the cause and date of death were obtained from the death certificate registry database maintained by the National Statistical Office of Korea. To prevent diagnostic inaccuracies, particularly overdiagnosis, we used only the primary discharge diagnosis as the study endpoint and excluded secondary diagnoses.

### 2.4. Statistical Analysis

Numbers are expressed as mean ± standard deviation or n (%). Student’s *t*-test was used for comparisons of continuous variables, and the Chi-square test was used for comparisons of non-continuous variables between the two groups. A multivariable Cox regression model was used to assess the independent association between SR carvedilol and MACE compared with IR carvedilol. In this multivariable analysis, models 1–4 were used sequentially to adjust for key clinical variables. The models adjusted for various covariates: model 1 adjusted for sex, age, income, smoking status, body mass index, systolic blood pressure, diastolic blood pressure, low-density lipoprotein cholesterol, high-density lipoprotein cholesterol, triglycerides, fasting glucose, and hemoglobin. Model 2 was additionally adjusted for the history of stroke, diabetes mellitus, dyslipidemia, myocardial infarction, hypertension, coronary artery disease, and heart failure in the past year, in addition to the adjustment variables of model 1. Models 3 and 4 further refined the adjustments by including medication adherence (proportion of days covered [PDC] ≥ 0.8) as a factor in models 1 and 2, respectively. PDC is the proportion of days in a given observation period that a patient has access to their prescribed medication, based on pharmacy refill data [15]. To assess whether the observed differences were attributable to real-world dosing patterns, patients receiving IR carvedilol were stratified by mean daily dosing frequency, using a prespecified cutoff of 1.5 doses/day. This threshold was chosen a priori as the midpoint between nominal once-daily (1.0) and recommended twice-daily (2.0) dosing, with values < 1.5 interpreted as approximating once-daily use and values ≥ 1.5 as more closely reflecting twice-daily administration. The mean daily dosing frequency of immediate-release carvedilol was calculated using the total number of prescribed tablets and the interval between outpatient prescription dates. Because this approach reflects real-world refill patterns, the resulting values did not fall exactly at 1 or 2 doses per day within individual patients. Therefore, rather than categorizing dosing strictly as once- or twice-daily, we prespecified a cutoff of 1.5 doses per day, corresponding to the midpoint between the nominal dosing frequencies. This threshold allowed differentiation between refill patterns approximating suboptimal once-daily use and those more consistent with recommended twice-daily administration. Statistical analyses were performed using SAS Software (version 9.4, SAS Institute, Cary, NC, USA) and R Statistical Software (version 4.0.3, R Foundation for Statistical Computing, Vienna, Austria).

## 3. Results

There were 6223 patients taking SR carvedilol and 32,340 patients taking IR carvedilol. The average daily carvedilol IR dose was 1.29 ± 0.43. Table 1 presents the baseline clinical characteristics of the study patients by drug type.

There were no significant differences in age, sex, or body mass index between patients with SR and those with IR receiving carvedilol. Both systolic blood pressure and diastolic blood pressure were significantly higher in patients with IR carvedilol than in those with SR carvedilol. Additionally, there were more current smokers in the IR carvedilol group compared to the SR carvedilol group. The SR carvedilol group had a higher prevalence of underlying diseases, including stroke, hypertension, diabetes mellitus, dyslipidemia, coronary artery disease, prior myocardial infarction, and heart failure. Analyzing income levels, the SR carvedilol group appeared somewhat better off than the IR carvedilol group. Blood test findings showed mostly no differences between the groups, although fasting glucose levels were slightly higher in the IR carvedilol group.

Clinical events that occurred during the median follow-up period of 730 days (698 ± 114 days for the carvedilol SR group and 709 ± 92 days for the carvedilol IR group; *p* for difference < 0.001) are presented in Table 2.

Among the total study population, 4561 (11.8%) experienced MACE. The rates of death, non-fatal myocardial infarction, heart failure requiring hospitalization, and MACE were significantly higher in patients taking IR carvedilol compared to those on SR carvedilol. The analysis presented in Table 3 details the hazard ratios (HRs) for various clinical outcomes comparing SR carvedilol to IR carvedilol.

The findings suggest a generally lower risk associated with SR carvedilol across multiple outcomes. For death, the HR in the crude model indicated a significantly lower risk with SR carvedilol; however, this association disappeared after adjusting for potential confounders. In the case of non-fatal myocardial infarction, SR carvedilol was associated with a significantly reduced risk in all models. The most adjusted model (Model 4) showed an HR of 0.54 (95% confidence interval [CI], 0.46–0.63; *p* < 0.001). For non-fatal stroke, no significant differences were observed between the SR and IR groups across all models, with HRs near 1, indicating no clear advantage of either formulation in preventing stroke. Heart failure requiring hospitalization was associated with a significantly lower risk with SR carvedilol across all models, with the HR in the most adjusted model (Model 4) being 0.53 (95% CI, 0.460–0.612; *p* < 0.001). Overall, MACE, which include death, non-fatal myocardial infarction, non-fatal stroke, and heart failure requiring admission, were significantly lower in patients using SR carvedilol. In Model 4, the HR for MACE was 0.61 (95% CI, 0.556–0.670, *p* < 0.001). When stratified by mean daily dosing frequency of IR carvedilol, patients receiving <1.5 doses/day had a significantly higher risk of MACE than those receiving SR carvedilol. In contrast, among patients with a mean daily dosing frequency ≥ 1.5, the difference in MACE risk between the IR and SR carvedilol groups was attenuated (Supplementary Table S1).

## 4. Discussion

In this large, real-world cohort study, we compared long-term clinical outcomes in patients prescribed SR or IR formulations of carvedilol. Despite differences in baseline characteristics, including higher blood pressure and smoking prevalence in the IR group and a greater burden of comorbidities in the SR group, our results consistently demonstrated more favorable cardiovascular outcomes with SR carvedilol. To the best of our knowledge, this is the first study to compare long-term cardiovascular outcomes between SR and IR formulations of carvedilol.

Carvedilol has established clinical benefits across cardiovascular conditions owing to its combined β- and α1-adrenergic blocking effects, which translate into favorable hemodynamic and cardioprotective actions. In the context of the present study, these pharmacological properties provide a biological basis for interpreting differences between formulations rather than reiterating their general efficacy. Sustained β-adrenergic blockade and vasodilatory effects are particularly relevant in chronic conditions such as heart failure and ischemic heart disease, where stable myocardial oxygen demand, reduced afterload, and attenuation of sympathetic activation are critical. Therefore, maintaining consistent drug exposure over the dosing interval, as intended with the SR formulation, may be more relevant to long-term outcomes than the intrinsic pharmacologic profile itself. This perspective supports interpreting our findings in terms of formulation-related exposure stability and real-world treatment patterns rather than revisiting the well-established mechanisms of carvedilol.

The development of an SR formulation of carvedilol was driven by the need to improve therapeutic outcomes and patient adherence in the long-term management of cardiovascular diseases. While IR carvedilol has been widely used and clinically effective, it requires twice-daily dosing due to its relatively short half-life [16]. This frequent dosing schedule can lead to fluctuations in plasma drug levels and presents challenges for patient compliance, particularly in elderly patients or those with complex medication regimens [10,11,17]. In our study, the average number of carvedilol IR doses taken per day, intended for twice-daily administration, was 1.29 doses. Nonadherence to medication is a well-documented issue in the treatment of chronic diseases such as hypertension, heart failure, and coronary artery disease [18,19,20]. Missing doses or inconsistent administration of carvedilol IR may compromise its β-blocking and vasodilatory effects, leading to suboptimal blood pressure control, increased risk of cardiovascular events, and poor clinical outcomes [20]. The SR formulation was therefore developed to maintain more stable plasma drug concentrations over a 24-h period, thereby enabling once-daily administration. Carvedilol SR offers several advantages over the IR formulation. Simplifying the dosing regimen enhances convenience and may significantly improve medication adherence [9,13]. Additionally, a more consistent pharmacokinetic profile minimizes peak-to-trough variability, potentially reducing side effects such as dizziness or fatigue that are sometimes observed with IR formulations. These improvements can translate into better tolerability and greater long-term efficacy, especially in populations requiring strict cardiovascular risk management [9,21].

In the stratified analysis, patients with a mean daily IR carvedilol dose < 1.5 had a significantly higher risk of MACE than those receiving SR carvedilol, even after adjustment, suggesting that suboptimal dosing or adherence may have contributed to outcome differences. This is consistent with our overall findings that the IR group had a higher incidence of MACE, likely attributable to a lower average dosing frequency. Adequate dosing appears to mitigate these differences, as patients taking IR carvedilol ≥ 1.5 times/day showed no significant difference in MACE risk compared with SR users. Because the 1.5-dose threshold was a pragmatic choice to differentiate patients whose refill patterns approximated once- versus twice-daily IR dosing, and alternative cut points could be considered, the subgroup findings based on dosing frequency should be interpreted as exploratory and hypothesis-generating rather than definitive.

Given that β-blockers such as carvedilol are integral to the management of heart failure, hypertension, and coronary artery disease, differences in dosing frequency and adherence could have influenced outcomes across multiple cardiovascular conditions. While a diagnosis-specific analysis could provide further insight, such stratification was limited by the overlapping comorbidities in our claims-based dataset. Although a more granular analysis stratified by primary diagnosis (e.g., hypertension, coronary artery disease, heart failure) would provide additional insight, such stratification was not feasible because cardiovascular comorbidities often overlap in many patients in the claims-based dataset. Because carvedilol is prescribed across multiple indications, including hypertension, coronary artery disease, and heart failure, differences in real-world dosing frequency and adherence may influence outcomes in a mixed cardiovascular population. In this nationwide claims-based cohort, these conditions often coexisted within the same individuals, and the dataset did not reliably identify a single primary indication or disease severity. Therefore, a diagnosis-specific comparative analysis could not be performed without introducing substantial misclassification or selection bias. While this inclusive design improves generalizability and reflects routine clinical practice, it also introduces clinical heterogeneity that may mask formulation-specific effects within individual subgroups. Accordingly, our findings should be interpreted as population-level associations rather than evidence of disease-specific comparative effectiveness. Future prospective studies or indication-focused analyses are warranted to clarify whether the associations between SR and IR carvedilol differ across hypertension, coronary artery disease, and heart failure populations.

Building on these pharmacokinetic advantages, carvedilol SR may confer additional pharmacodynamic benefits, translating into superior clinical outcomes compared to the IR formulation. By maintaining steady plasma drug concentrations, SR carvedilol ensures continuous β-adrenergic and α1-adrenergic blockade, which may enhance blood pressure stability and reduce myocardial oxygen demand throughout the dosing interval. Moreover, the prevention of abrupt declines in drug levels could limit rebound sympathetic activation, a critical factor in exacerbations of ischemia and heart failure. In this study, patients receiving SR carvedilol demonstrated significantly lower rates of MACE, non-fatal myocardial infarction, and heart failure hospitalizations, despite having a greater baseline burden of comorbidities. These findings suggest that sustained pharmacologic coverage and improved patient adherence associated with SR carvedilol contributed meaningfully to the observed improvements in long-term cardiovascular outcomes.

In interpreting our findings, it is important to consider current heart failure guidelines, which typically recommend the IR formulation of carvedilol for patients with HFrEF, usually administered twice daily [22]. This suggests that the IR group in our study may have included a higher proportion of patients with HFrEF, which could have predisposed them to worse outcomes regardless of formulation type. However, in our cohort, the prevalence of a coded heart failure diagnosis was actually higher in the SR group than in the IR group (15.8% vs. 9.1%, *p* < 0.001). This suggests that SR carvedilol was, in practice, more frequently prescribed to patients with documented heart failure, which would generally be expected to bias results toward worse outcomes in the SR group. We acknowledge that misclassification and limitations of administrative coding may obscure the true prevalence and severity of HFrEF in each group, and this remains a potential source of residual confounding. Nonetheless, given that the SR group also had higher rates of other comorbidities such as prior myocardial infarction, coronary artery disease, diabetes mellitus, and dyslipidemia, the consistently more favorable outcomes observed with SR carvedilol are unlikely to be fully explained by baseline differences in heart failure burden alone.

Several limitations of this study should be acknowledged. First, the retrospective observational design inherently introduces the possibility of residual confounding, despite adjustment for a wide range of baseline clinical characteristics. More advanced statistical approaches, such as propensity score matching or inverse probability of treatment weighting, would have further strengthened causal inference; however, due to the inherent constraints of the NHIS database and data access environment, we were unable to conduct these analyses. Important unmeasured variables, such as patient adherence beyond prescription refills, precise heart failure severity (e.g., ejection fraction, N-terminal pro-brain natriuretic peptide levels), or specific details of comorbid conditions, may have influenced outcomes. The mean follow-up duration was statistically shorter in the SR carvedilol group than in the IR carvedilol group, which could introduce differential exposure time and potentially bias incidence estimates, thereby partially contributing to the lower observed incidence of MACE in the SR group. Although the absolute difference in follow-up duration was modest (11 days), and our time-to-event analyses (person–time denominators and Cox proportional hazards models) inherently account for variable follow-up, residual bias related to unequal exposure time cannot be completely excluded. Additional sensitivity analyses, such as time-dependent exposure modeling or landmark analyses, could further address this concern; however, these were not feasible within the constraints of the claims-based dataset and our predefined exposure definition (treatment initiation, excluding formulation crossover or prolonged discontinuation). Therefore, the findings should be interpreted cautiously, and prospective studies with finer-grained longitudinal exposure data are warranted to confirm these observations. Second, although we used a large, real-world cohort based on prescription and claims data, misclassification bias remains possible, as diagnostic codes (ICD-10) may not fully capture clinical nuances or severity. Third, the NHIS-NHID does not provide cause-specific mortality, such as cardiac death, in its standard dataset; therefore, all-cause death was included in the composite MACE definition. This approach is consistent with prior large-scale observational studies using administrative data, as demonstrated by a recent meta-analysis showing that all-cause mortality is commonly used when cause-specific mortality data are unavailable [23]. Fourth, although we adjusted for medication adherence using a predefined PDC threshold (≥0.8), we were unable to present detailed PDC distributions separately for the SR and IR carvedilol groups, and we could not assess direct adherence behaviors (e.g., timing, actual ingestion). In addition, differences in socioeconomic factors, health-seeking behavior, or physician prescribing preferences between SR and IR carvedilol users might have contributed to the observed outcome differences but were not fully controlled for. Fifth, the study population consisted of Korean patients, which may limit the generalizability of the findings to other ethnic or healthcare system contexts. In addition, this study did not focus exclusively on patients with hypertension, coronary artery disease, or heart failure, which may limit the ability to draw disease-specific conclusions. Finally, information on side effects, tolerability, and quality of life, important factors influencing long-term β-blocker therapy, was not available in the dataset. Additionally, the lack of information on concomitant cardioprotective therapies such as renin–angiotensin system inhibitors, sodium–glucose cotransporter 2 inhibitors, angiotensin receptor–neprilysin inhibitors, antiplatelet agents, and statins represents another important limitation. Future prospective randomized controlled trials are needed to confirm the clinical benefits of SR carvedilol observed in this study.

## 5. Conclusions

In this large, real-world claims-based cohort study, SR carvedilol was associated with more favorable long-term clinical outcomes than IR carvedilol, including lower rates of MACE, non-fatal myocardial infarction, and heart failure hospitalization. However, these findings should be interpreted as associations rather than evidence of causal superiority, as selection bias and residual confounding may remain. The potential contributions of pharmacokinetic differences and adherence are plausible but cannot be confirmed in this study. Prospective randomized studies are warranted to validate these observations and to clarify the role of SR formulations in cardiovascular disease management.

## Figures and Tables

**Table 1:** Baseline clinical characteristics of study patients.

Characteristic	SR Carvedilol (*n* = 6223)	IR Carvedilol (*n* = 32,340)	*p*
Age, years	54.8 ± 11.7	54.9 ± 12.1	0.875
Female sex	2062 (33.1)	10,697 (33.0)	0.929
Body mass index, kg/m^2^	25.1 ± 3.3	25.2 ± 3.5	0.473
Systolic blood pressure, mmHg	131 ± 18	133 ± 20	<0.001
Diastolic blood pressure, mmHg	82.4 ± 13.3	83.4 ± 13.6	<0.001
*Smoking status*			
Non-smoker	2952 (47.4)	15,106 (46.7)	<0.001
Ex-smoker	1371 (22.0)	6543 (20.2)	
Current smoker	1900 (30.5)	10,691 (33.0)	
*Underlying disease*			
Stroke	431 (6.93)	1757 (5.43)	<0.001
Hypertension	3345 (53.7)	16,681 (51.5)	0.002
Diabetes mellitus	1552 (24.9)	6720 (20.7)	<0.001
Dyslipidemia	3622 (58.2)	15,275 (47.2)	<0.001
Coronary artery disease	771 (12.8)	1773 (5.4)	<0.001
Prior myocardial infarction	642 (10.3)	1728 (5.3)	<0.001
Heart failure	988 (15.8)	2969 (9.1)	<0.001
*Income level*			
The first quartile (the lowest)	1161 (18.6)	7069 (21.8)	<0.001
The second quartile	1199 (19.2)	6471 (20.0)	
The third quartile	1590 (25.5)	8641 (26.7)	
The fourth quartile (the highest)	2273 (36.5)	10,159 (31.4)	
*Laboratory findings*			
Total cholesterol, mg/dL	207 ± 41	207 ± 43	0.300
LDL cholesterol, mg/dL	124 ± 39	124 ± 46	0.544
HDL cholesterol, mg/dL	52 ± 13	52.4 ± 14.8	0.507
Triglycerides, mg/dL	160 ± 123	162 ± 125	0.148
Fasting glucose, mg/dL	105 ± 30	106 ± 33	0.005
Hemoglobin, g/dL	14.6 ± 1.6	14.6 ± 1.7	0.056

Numbers are expressed as mean ± standard deviation or *n* (%). SR, slow release; IR, immediate release; LDL, low-density lipoprotein; HDL, high-density lipoprotein;

**Table 2:** Clinical events.

Clinical Event	SR Carvedilol (*n* = 6223)	IR Carvedilol (*n* = 32,340)	*p*
*Death*			
N of event (incidence)	47 (0.7)	338 (1.0)	0.035
Person-year	12,407	64,368	
Rate (per 100,000)	379	525	
*Non-fatal myocardial infarction*			
N of event (incidence)	212 (3.4)	1562 (4.8)	<0.001
Person-year	12,285	63,351	
Rate (per 100,000)	1726	2466	
*Non-fatal stroke*			
N of event (incidence)	133 (2.1)	720 (2.2)	0.662
Person-year	12,349	64,034	
Rate (per 100,000)	1077	1124	
*Heart failure requiring hospitalization*			
N of event (incidence)	234 (3.7)	1947 (6.0)	<0.001
Person-year	12,253	63,015	
Rate (per 100,000)	1910	3090	
MACE *			
N of event (incidence)	554 (8.9)	4007 (12.3)	<0.001
Person-year	12,203	61,887	
Rate (per 100,000)	4577	6475	

Numbers are expressed as n (%). * MACE includes death, non-fatal myocardial infarction, non-fatal stroke, heart failure requiring hospitalization. SR, slow release; IR, immediate release; MACE, major adverse cardiovascular event.

**Table 3:** The hazard ratio for SR carvedilol compared to IR carvedilol with respect to clinical outcomes.

	Death		Non-Fatal MI		Non-Fatal Stroke		HF Hospitalization		MACE *	
	HR (95% CI)	*p*	HR (95% CI)	*p*	HR (95% CI)	*p*	HR (95% CI)	*p*	HR (95% CI)	*p*
Crude	0.72 (0.53–0.97)	0.036	0.69 (0.60–0.80)	<0.001	0.95 (0.79–1.15)	0.643	0.61 (0.53–0.70)	<0.001	0.70 (0.64–0.76)	<0.001
Model 1	0.79 (0.58–1.08)	0.149	0.69 (0.60–0.79)	<0.001	1.00 (0.83–1.20)	0.993	0.61 (0.53–0.70)	<0.001	0.70 (0.64–0.76)	<0.001
Model 2	0.77 (0.57–1.06)	0.114	0.54 (0.46–0.62)	<0.001	0.92 (0.76–1.11)	0.430	0.51 (0.44–0.58)	<0.001	0.60 (0.54–0.65)	<0.001
Model 3	0.79 (0.57–1.11)	0.180	0.67 (0.58–0.78)	<0.001	1.04 (0.86–1.26)	0.643	0.62 (0.54–0.71)	<0.001	0.70 (0.64–0.77)	<0.001
Model 4	0.78 (0.56–1.08)	0.145	0.54 (0.46–0.63)	<0.001	0.97 (0.80–1.18)	0.794	0.53 (0.46–0.61)	<0.001	0.61 (0.55–0.67)	<0.001

Model 1 was adjusted by sex, age, income, smoking status, body mass index, systolic blood pressure, diastolic blood pressure, low-density lipoprotein cholesterol, high-density lipoprotein cholesterol, triglycerides, fasting glucose, and hemoglobin. Model 2 was additionally adjusted for the history of stroke, diabetes mellitus, dyslipidemia, myocardial infarction, hypertension, coronary artery disease, and heart failure in the past year, in addition to the adjustment variables of Model 1. Model 3 was adjusted for sex, age, income, smoking status, body mass index, systolic blood pressure, diastolic blood pressure, low-density lipoprotein cholesterol, high-density lipoprotein cholesterol, triglycerides, fasting glucose, and hemoglobin, including participants whose proportion of days covered was ≥0.8. Model 4 was additionally adjusted for the history of stroke, diabetes, dyslipidemia, myocardial infarction, hypertension, coronary artery disease, and heart failure in the past year, in addition to the adjustment variables of Model 3, including participants whose proportion of days covered was ≥0.8. * MACE includes death, non-fatal myocardial infarction, non-fatal stroke and heart failure requiring admission. SR, slow release; IR, immediate release; MI, myocardial infarction; HF, heart failure; MACE, major adverse cardiovascular event; HR, hazard ratio; CI, confidence interval.

## Data Availability

The KNHIS database used in this study is available to Korean researchers who submit a research proposal in accordance with the prescribed procedures and obtain approval from the relevant authority.

## Supplementary Materials

The following supporting information can be downloaded at: https://www.mdpi.com/article/10.3390/jcm15041417/s1, Table S1: The hazard ratio for SR carvedilol compared to IR carvedilol with respect to clinical outcomes.
