# Supplementary material for: Comparison of Long-Term Clinical Outcomes Between Immediate-Release and Slow-Release Carvedilol: A National Real-World Database Analysis
Source: J Clin Med. 2026 Feb 11;15(4):1417. doi: 10.3390/jcm15041417 (PMC12941224; doi:10.3390/jcm15041417)
Supplement: Supplementary file 1 [file jcm-15-01417-s001.zip › jcm-4132685-supplementary.pdf]

Supplementary Table S1. The hazard ratio for SR carvedilol compared to IR carvedilol with respect to clinical outcomes

	Death		Non-fatal MI		Non-fatal stroke		HF hospitalization		MACE*	
	**HR (95% CI)**	***P***	**HR (95% CI)**	***P***	**HR (95% CI)**	***P***	**HR (95% CI)**	***P***	**HR (95% CI)**	***P***
In patients with a mean daily dosing frequency of IR carvedilol < 1.5										
**Crude**	0.44 (0.32–0.61)	< 0.001	0.36 (0.31–0.42)	< 0.001	0.65 (0.53–0.80)	< 0.001	0.28 (0.25–0.33)	< 0.001	0.35 (0.32–0.38)	< 0.001
**Model 1**	0.60 (0.43–0.83)	0.002	0.43 (0.37–0.50)	< 0.001	0.73 (0.59–0.90)	0.003	0.31 (0.27–0.36)	< 0.001	0.40 (0.36–0.44)	< 0.001
**Model 2**	0.60 (0.43–0.84)	0.003	0.40 (0.34–0.46)	< 0.001	0.663 (0.53–0.81)	< 0.001	0.32 (0.28–0.37)	< 0.001	0.39 (0.35–0.43)	< 0.001
In patients with a mean daily dosing frequency of IR carvedilol ≥ 1.5										
**Crude**	0.97 (0.70–1.33)	0.846	1.09 (0.94–1.28)	0.232	1.17 (0.96–1.43)	0.101	1.11 (0.96–1.28)	0.149	1.12 (1.01–1.23)	0.019
**Model 1**	0.95 (0.68–1.31)	0.759	0.95 (0.81–1.11)	0.568	1.15 (0.95–1.41)	0.141	1.01 (0.87–1.17)	0.891	1.01 (0.92–1.11)	0.732
**Model 2**	0.92 (0.66–1.28)	0.626	0.65 (0.56–0.77)	< 0.001	1.07 (0.88–1.31)	0.467	0.74 (0.63–0.86)	< 0.001	0.79 (0.72–0.87)	< 0.001

Model 1 was adjusted by sex, age, income, smoking status, body mass index, systolic blood pressure, diastolic blood pressure, low-density lipoprotein cholesterol, high-density lipoprotein cholesterol, triglycerides, fasting glucose, and hemoglobin.

Model 2 was additionally adjusted for the history of stroke, diabetes mellitus, dyslipidemia, myocardial infarction, hypertension, coronary artery disease, and heart failure in the past year, in addition to the adjustment variables of Model 1

*MACE includes death, non-fatal myocardial infarction, non-fatal stroke and heart failure requiring admission.

SR, sustained release; IR, immediate release; MI, myocardial infarction; HF, heart failure; MACE, major adverse cardiovascular event; HR, hazard ratio; CI, confidence interval.
